# Pre-engraftment neurological impairment in allogeneic stem cell transplant: A case report of atypical posterior reversible encephalopathy syndrome with pontine involvement

**DOI:** 10.3389/frtra.2022.1089995

**Published:** 2023-01-25

**Authors:** Andrea Acerbis, Giorgio Orofino, Edoardo Campodonico, Anna Del Poggio, Elisabetta Xue, Francesca di Matteo, Greta Spelta, Alessandro Bruno, Andrea Falini, Fabio Ciceri, Jacopo Peccatori, Raffaella Greco

**Affiliations:** ^1^ Hematology and Bone Marrow Transplant Unit, IRCCS San Raffaele Hospital, Milan, Italy; ^2^ Vita-Salute San Raffaele University, Milan, Italy; ^3^ Department of Neuroradiology and CERMAC, San Raffaele Hospital, Milan, Italy

**Keywords:** PRES—posterior reversible encephalopathy syndrome, bone marrow translation (BMT), allogeneic, neuroimaging, neurological complications

## Abstract

In the present report, we describe the case of a 59-year-old female who developed pre-engraftment multiple organ failure (MOF) after allogeneic hematopoietic stem cell transplant (HSCT), followed a few days later by a cohort of neurological symptoms leading to a diagnosis of posterior reversible encephalopathy syndrome (PRES). The diagnosis was achieved by excluding more frequent entities associated with neurological symptoms in HSCT and supported by compatible magnetic resonance imaging (MRI) findings, with remarkably interesting less frequent pontine involvement. GvHD prophylaxis, including sirolimus and mycophenolate mofetil (MMF), was discontinued, while carefully controlling blood pressure. In addition, high-dose steroids were employed. After 2 weeks, the neurological symptoms abated, and follow-up MRI showed a complete regression of neurological alterations, confirming the diagnostic hypothesis of PRES.

## Introduction

Allogeneic hematopoietic stem cell transplantation (HSCT) represents the standard curative treatment for several malignant and non-malignant hematological disorders (1). Transplant outcomes have steadily improved throughout the last decade, thanks to better patient selection, well-tolerated reduced-toxicity conditioning (RTC), and improvement in supportive care (2).

**Figure 1:**
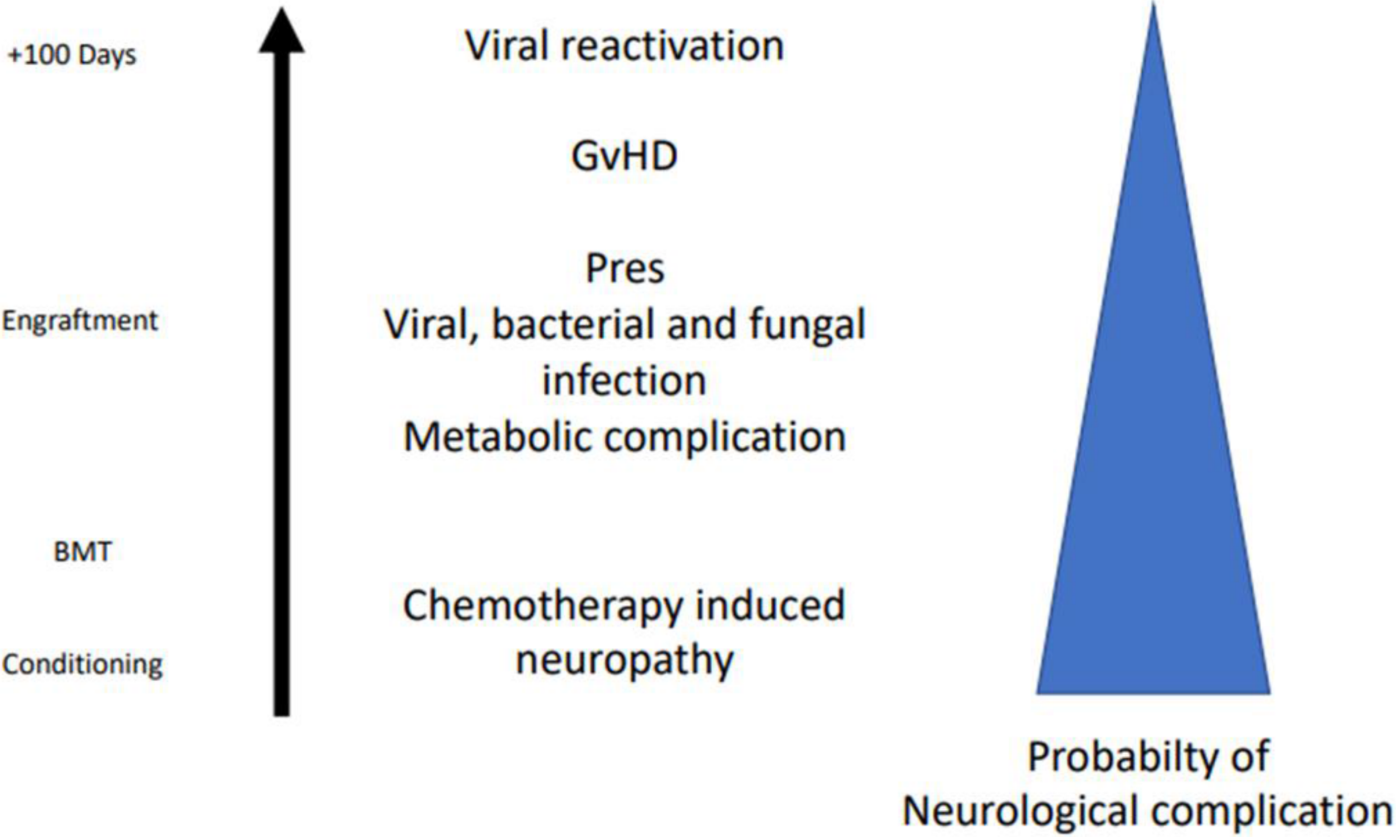
Possible etiologies of neurological complications at different time points (during conditioning, engraftment phase, and after 100 days) in the setting of allogeneic HSCT. BMT, bone marrow transplantation; GvHD, graft versus host disease; Pres, posterior reversible encephalopathy.

Neurological complications following allogeneic HSCT are not infrequent, may have protean clinical manifestations, and are often difficult to identify promptly (Figure 1) (3).

**Figure 2:**
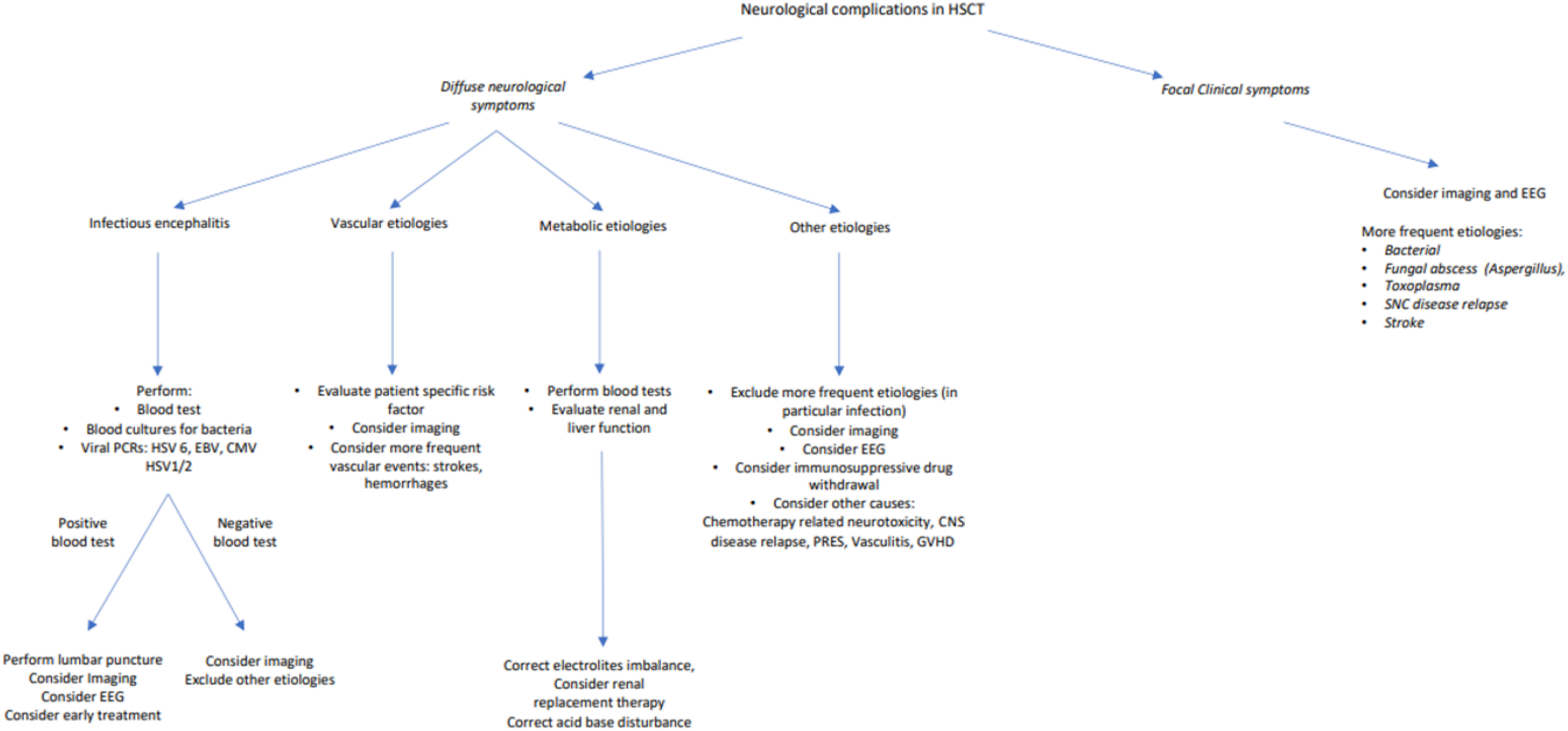
Possible differential diagnosis and diagnostic algorithm of neurological complications in patients undergoing allogeneic HSCT. PCR, polymerase chain reaction; HSV 6, human herpes virus 6; EBV, Epstein–Barr virus; CMV, cytomegalovirus; HSV 1/2, human herpes virus 1/2; EEG, electroencephalogram; CNS, central nervous system.

The vast majority of neurological syndromes complicating HSCT are infectious, drug-related, metabolic, vascular, or immune-mediated in nature (Figure 2). Among the causes of infections of encephalitis, HHV-6, VZV, EBV, CMV, HSV-1 and 2, adenovirus, enterovirus, and more recently, SARS-CoV-2 (Figure 2), are the most frequent ones, although bacterial abscess and fungal meningitis must be taken into consideration. A careful review of the medication history is essential, because chemotherapeutic agents used in conditioning regimens (i.e., fludarabine) and also drugs serving as graft-versus-host-disease (GvHD) prophylaxis (i.e., sirolimus and cyclosporine) may cause neurotoxicity. Metabolic encephalopathy, cerebrovascular illness, and immune-mediated diseases must also enter the physician's differential diagnosis (4). After more frequent etiologies are ruled out, posterior reversible encephalopathy syndrome (PRES) may be considered (3).

PRES is a clinical-radiographic entity caused by heterogeneous etiologies, showing similar findings on neuroimaging studies and a common series of clinical symptoms.

The most common underlying causes related to this syndrome are hypertensive encephalopathy, eclampsia, and the use of cytotoxic and immunosuppressive drugs. In particular, PRES often occurs in patients treated with immunosuppressive drugs after allogeneic transplant, such as cyclosporine, methotrexate, sirolimus, and tacrolimus, and also in cancer patients treated with platinum-containing drugs, CHOP regimens, and gemcitabine (5).

PRES is caused by acute fluid extravasation (vasogenic edema), typically in the subcortical parieto-occipital white matter (hence the term “posterior”), and is usually reversible.

Pathogenesis is complex and includes impaired vascular autoregulation and/or endothelial injury. An alteration of the normal autoregulation of the cerebral blood flow may be the cause, with cerebral blood flow fluctuations that exceed the limits of cerebral autoregulation with hyper-/hypoperfusion and breakthrough vasogenic edema. Another etiologic hypothesis may be endothelial dysfunction and altered permeability, which are related to the condition of sepsis, immunesuppressive treatments, and eclampsia, combined with conditions of capillary leakage and blood–brain barrier disruption (6).

Clinically, the typical PRES symptoms are headache, altered consciousness, visual disturbances (hemianopia, visual neglect, auras, visual hallucinations, and cortical blindness), and seizure, which are often the presenting manifestations (6).

Typically, on imaging, vasogenic edema develops in patchy or confluent areas of subcortical parieto-occipital white matter with a variable involvement of the adjacent gray matter. Usually, PRES involvement is bilateral and symmetric, although asymmetry is not uncommon. PRES could also involve frontal and, rarely, temporal regions, and less frequently, may also involve the basal ganglia, cerebellum, and brainstem.

There are no specific diagnostic clinical criteria for PRES, which remains a diagnosis of exclusion: for this reason, in appropriate clinical settings, clinicians must be guided by the presence of the typical symptoms and MRI findings, which are important to support the diagnosis.

MRI with gadolinium is the investigation of choice when PRES is clinically suspected. Brain imaging with non-enhanced CT (NECT) is performed often as first-line approach to rule out the presence of ischemic/hemorrhagic stroke or intracranial masses that may justify acute symptoms. Areas of vasogenic edema appear hypodense on NECT. Different from stroke, the subcortical areas are much more involved than the cortex. To enable patients achieve a low sensitivity to CT, neuroimaging with MRI is mandatory in diagnosis. The most informative MRI sequences are T2-FLAIR, which shows focal or confluent areas of increased signals, and diffusion-weighted imaging with an apparent diffusion coefficient map (DWI-ADC), which shows a hypo- or isointense signal in DWI with high ADC values, consistent with vasogenic edema (7).

Diagnosis must be timely, because PRES is a reversible condition with a benign prognosis if appropriate management is done. The key elements of treatment are a gradual blood pressure lowering, medications for seizure prevention (if seizure occurs), and a discontinuation of immunosuppressive therapy.

## Case presentation

We report the case of a 59-year-old female patient with unremarkable past medical history, diagnosed with 46(XX), NPM1 wild-type, and FLT3 wild-type acute myeloid leukemia. She underwent bone marrow transplantation after achieving a second complete remission with five cycles of Decitabine-Venetoclax. A 4/6 cord blood unit (CBU) was selected as the source of hematopoietic stem cells. The conditioning regimen included treosulfan (14.000 mg/m^2^ for 3 days) and fludarabine (30 mg/m^2^ for 5 days) (8).

GvHD prophylaxis was based on sirolimus (1 mg once daily) from day 7 and mycophenolate mofetil (10 mg/kg twice daily) from day 1 as per our institutional protocol (9, 10).

Until day 59 after bone marrow transplantation, neutrophil counts remained consistently below 500 cells/mm^3^ and the patient remained dependent on red blood cell and platelet transfusion. A bone marrow aspiration was performed on day 30, showing a polymerase chain reaction (PCR) chimerism with only a 3.4% host signal, suggesting a poor graft function rather than a transplant rejection.

In this pre-engraftment phase, the woman experienced several events, including multi-drug-resistant (MDR) *P. aeruginosa* infection and probable invasive aspergillosis. A longitudinal assessment for SARS-CoV-2 by using a nasopharyngeal swab was performed, and no infection was documented. As a consequence, the clinical conditions rapidly worsened and she developed multiple organ failure (MOF), including acute kidney injury (AKI) requiring continuous veno-venous hemodialysis (CVVH) and acute hepatic failure and respiratory failure requiring high-flow oxygen therapy and non-invasive ventilation. At this point, the patient was alert, oriented, and had neither focal deficits nor cognitive impairment.

**Figure 3:**
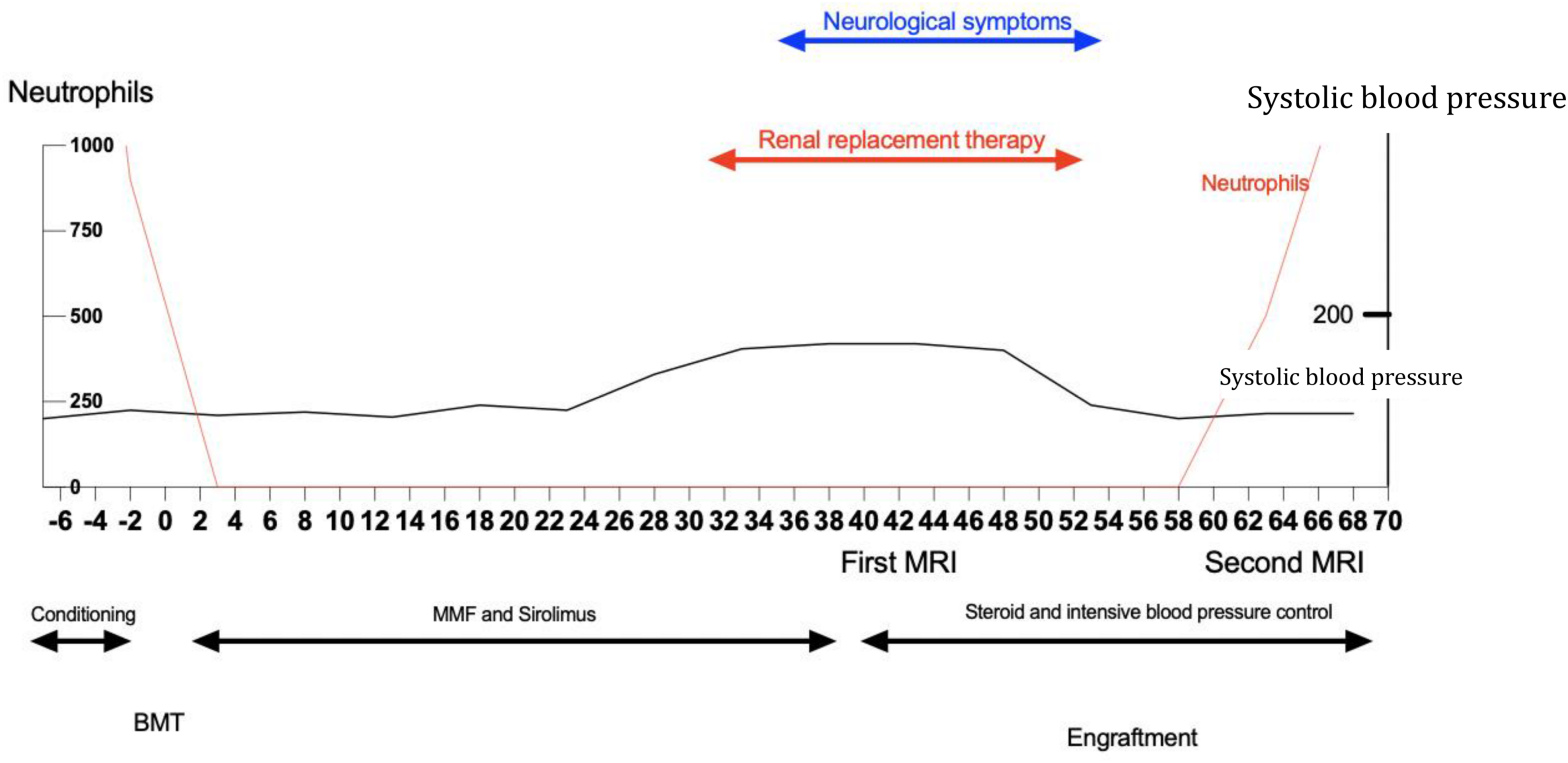
Schematic representation of the case. BMT, bone marrow transplant; MRI, magnetic resonance imaging; MMF, mycophenolate mofetil.

Five days after the onset of MOF, the patient started to recover. At the same time, new neurological symptoms appeared, starting with tremors and mild confusion. Her blood pressure levels rose up to 190/100 mmHg (Figure 3). Neurological symptoms worsened quickly, leading to altered consciousness, ataxia, and optic phosphenes. The Glasgow Coma Scale (GCS) score dropped from normal to 8 out of 15.

In our clinical case, cytomegalovirus (CMV), Epstein–Barr virus (EBV), and herpes virus-1, 2, 3, and 6 were analyzed in the peripheral blood, and no reactivations were observed. Due to a lack of peripheral blood viral reactivation, a low clinical and radiological suspicion for viral infection, and the critical condition of the patient, lumbar puncture was not performed.

A brain CT scan showed a nuanced hypodensity in the cortical–subcortical occipital region, mainly in the right lobe, which was suspicious of an ischemic lesion. Electroencephalogram (EEG) documented an unspecific alteration of the general organization, symmetrically expressed in both cerebral hemispheres. Moreover, some focal slow abnormalities were present.

Given the post-transplant timing and the complex clinical picture (AKI, evidence of hepatic insufficiency with an elevation of necrosis markers, and an aspecificity of neuroimaging and EEG), our first hypothesis focused on metabolic encephalopathy, although transplant-related microangiopathy could not be excluded. Sirolimus was interrupted in favor of a high-dose intravenous methylprednisolone (Figure 3).

Although we observed a progressive normalization of renal and hepatic function, no neurological *restitutio ad integrum* (restoration to original condition) occurred.

**Figure 4:**
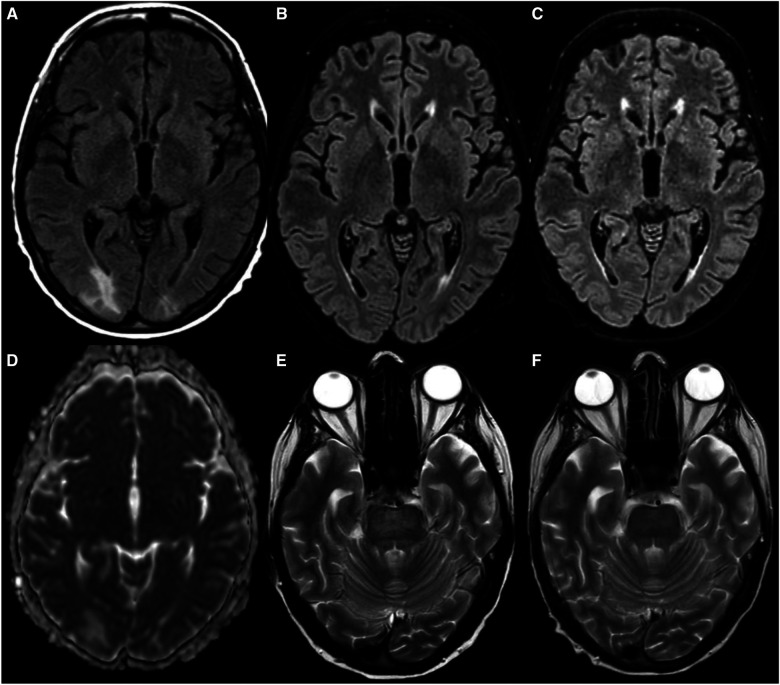
Brain MRI: (**A–C**) FLAIR images; (**D**) ADC map; (**E,F**) T2 weighted images. (**A,D**) first MRI shows areas of a hyperintense signal in FLAIR at the level of the occipital poles bilaterally with a greater extension to the right one with high AD values consistent with edema; (**B,E**): second MRI shows a resolution of FLAIR hyperintensities referable to vasogenic edema in the bilateral polar occipital cortico-subcortical area; on the other hand, diffuse centropontine alteration in T2/FLAIR sequences (FLAIR not shown) appears; (**C,F**) third MRI shows a complete regression of the whole alteration both in the supratentorial posterior white matter and in the pons. MRI, magnetic resonance imaging; FLAIR, fluid attenuated inversion recovery; ADC map, apparent diffusion coefficient map; AD value, ADded value.

The first brain MRI analysis (Figure 4) revealed a hyperintensity area in T2-FLAIR sequences, in both occipital hemispheres. The use of multiple MRI scans, including diffusion-weighted imaging, suggested a plausible etiology for the neurological impairment and later demonstrated the progressive involvement of different areas in the brain. The areas of signal alteration were initially mainly localized near the subcortical white matter with minimal involvement of the cortex, primarily in the right occipital hemisphere. Due to the low likelihood of viral infection and after ruling out ischemic and drug-induced encephalopathy, considering compatible symptoms and imaging findings, we hypothesized the diagnosis of PRES, which, as previously mentioned, is an exclusion diagnosis, leading to a more stringent blood-pressure control. High-dose steroids were maintained, keeping sirolimus under discontinuation mode. Gradually, neurological symptoms ameliorated, reaching a state of complete recovery 14 days after onset. We empirically confirmed the diagnosis of PRES in the light of complete symptom regression.

Two weeks later, on day 59, neutrophil engraftment was observed from HSCT (Figure 3). Donor chimerism was analyzed by using PCR at different time points, on day 60 and on day 120 post-transplant, showing full donor chimerism.

One month later, we performed a second brain MRI showing a complete recovery of previously reported abnormalities; while occipital signal alteration disappeared, a new area of hyperintensity was detected in the pontine areas (Figure 4).

This is a rare but described finding during PRES progression, but it could be also a sign of central pontine myelinolysis (11). However, the ADC value was high, which is consistent with edema. However, in osmotic myelinolysis, diffusion of water would be restricted with low ADC values. Moreover, since the electrolyte values were normal and there were no pontine-related symptoms, central pontine myelinolysis was ruled out.

The patient underwent a third brain MRI (Figure 4) one and a half months after the second one, which showed a regression of signal abnormalities in the pons, a finding consistent with the diagnosis of PRES.

The patient experienced a progressive improvement and no further neurological symptoms were reported: she was discharged on day 133, and follow-up treatment was continued on an outpatient basis.

## Discussion

This study describes the case of a patient affected by PRES after allogeneic HSCT before neutrophil engraftment. This case represents a good basis for a discussion of the differential diagnosis of neurological symptoms in the setting of HSCT.

When new-onset neurological deficits ensue after HSCT, it is mandatory to perform a proper differential diagnosis, which revolves around four main criteria: timing, clinical presentation, imaging findings, and biochemical alterations.

First and foremost, the time of onset may suggest the most plausible cause (12). Toxicities related to the conditioning regimen typically occur relatively early after transplant. Bacterial and fungal infections and cerebrovascular accidents (both ischemic and hemorrhagic) are especially frequent before and soon after engraftment (13). Conversely, corticosteroid and immunosuppressant toxicity, transplant-related microangiopathy, and herpetic reactivation are more relevant after engraftment (4). A chronic GvHD of the brain, although anecdotal and controversial, may also occur as a late-onset neurological complication after HSCT. Due to many confounding factors related to the HSCT procedure, such as drugs used in conditioning regimens, immunosuppressants, and infections of the CNS that are associated with an increased risk of neurocognitive toxicities, it has become difficult to distinguish the direct effects of GvHD on the CNS from those resulting from other complications of HSCT. According to the National Institutes of Health Consensus on Criteria for Clinical Trials in Chronic GvHD, a diagnosis of chronic GvHD of the nervous system can be made only when other organs are affected by GvHD and frequent neurological differential diagnoses such as drug-induced toxicities or opportunistic infections are excluded (14). Among the viral causes, it is crucial to rule out HHV-6 encephalitis, especially in the context of cord blood unit and haploidentical transplantation treated with post-transplantation cyclophosphamide (15).

With regard to the clinical presentation, it is relevant to keep in mind that different etiologies might be harbored by distinct clinical pictures, depending on the brain area most frequently affected: PRES is typically related to an occipital involvement, with confusion and visual alterations, while viral encephalitis might be revealed by subtle behavioral changes due to the fronto-temporal involvement of MRI. In this context, brain MRI may be an important additional tool in the diagnostic flow for ruling out post-transplant CNS complications.

Finally, complementary laboratory investigations are an invaluable tool to orient clinicians toward a diagnosis. A concomitant detection of schistocytes on the peripheral blood smear, platelet-refractoriness, and renal damage strongly makes a case for transplant-associated microangiopathy or immune suppressant–induced neurotoxicity. Conversely, peripheral reactivations of herpes virus-1, -6, or other viruses might hint at an ongoing viral encephalitis. In this context, lumbar puncture is particularly useful to rule out infectious etiologies; however, this procedure might be challenging to perform in the setting of post-transplant thrombocytopenia.

To conclude, in the first 100 days after HSCT, we cannot overemphasize the importance of a multidisciplinary discussion: indeed, in our case, the cooperation of the radiologist, infectious disease specialist, and neurologist was pivotal to arrive at a correct diagnosis. Neurological symptoms may be extremely non-specific in HSCT patients, imaging and diagnostic tests may be falsely negative, and expertise in HSCT is required even in non-hematologic consultants for such patients.

## Data Availability

The raw data supporting the conclusions of this article will be made available by the authors without undue reservation.
